# An Effective Approach to Improve the Automatic Segmentation and Classification Accuracy of Brain Metastasis by Combining Multi-phase Delay Enhanced MR Images

**DOI:** 10.1007/s10278-023-00856-3

**Published:** 2023-05-31

**Authors:** Mingming Chen, Yujie Guo, Pengcheng Wang, Qi Chen, Lu Bai, Shaobin Wang, Ya Su, Lizhen Wang, Guanzhong Gong

**Affiliations:** 1 Department of Radiation Physics, Shandong First Medical University Affiliated Cancer Hospital, Shandong Cancer Hospital and Institute (Shandong Cancer Hospital), Jinan, 250117 China; 2 College of Radiology, Shandong First Medical University & Shandong Academy of Medical Sciences, Jinan, 250117 China; 3 Department of Engineering Physics, Tsing Hua University, Beijing, 100084 China; 4 MedMind Technology Co., Ltd, 100084 Beijing, China

**Keywords:** Brain metastasis, Magnetic resonance imaging, Contrast media, Classification, Segmentation

## Abstract

The objective of this study is to analyse the diffusion rule of the contrast media in multi-phase delayed enhanced magnetic resonance (MR) T1 images using radiomics and to construct an automatic classification and segmentation model of brain metastases (BM) based on support vector machine (SVM) and Dpn-UNet. A total of 189 BM patients with 1047 metastases were enrolled. Contrast-enhanced MR images were obtained at 1, 3, 5, 10, 18, and 20 min following contrast medium injection. The tumour target volume was delineated, and the radiomics features were extracted and analysed. BM segmentation and classification models in the MR images with different enhancement phases were constructed using Dpn-UNet and SVM, and differences in the BM segmentation and classification models with different enhancement times were compared. (1) The signal intensity for BM decreased with time delay and peaked at 3 min. (2) Among the 144 optimal radiomics features, 22 showed strong correlation with time (highest *R*-value = 0.82), while 41 showed strong correlation with volume (highest *R*-value = 0.99). (3) The average dice similarity coefficients of both the training and test sets were the highest at 10 min for the automatic segmentation of BM, reaching 0.92 and 0.82, respectively. (4) The areas under the curve (AUCs) for the classification of BM pathology type applying single-phase MRI was the highest at 10 min, reaching 0.674. The AUC for the classification of BM by applying the six-phase image combination was the highest, reaching 0.9596, and improved by 42.3% compared with that by applying single-phase images at 10 min. The dynamic changes of contrast media diffusion in BM can be reflected by multi-phase delayed enhancement based on radiomics, which can more objectively reflect the pathological types and significantly improve the accuracy of BM segmentation and classification.

## Introduction

The incidence of brain metastasis (BM) of primary malignant tumours, common in lung cancer, breast cancer, melanoma, and gastrointestinal adenocarcinoma, has reached 80% [1–3]. With advancement in treatment strategies, patient survival has significantly prolonged, and hence, the incidence of metastases has increased; thus, BM has become the primary cause of malignant tumour treatment failure [4, 5]. However, in about 15–20% of cases, BM was the first symptom of the patient, and the primary tumour cannot be identified [6]. By identifying pathological types early in patients with brain-first symptoms, we can accurately determine the primary tumour, formulate individualised treatment plans, and segment the tumour accurately. Previous studies have also proved that most of the patients with BM with an unknown primary focus had poor prognosis, which demonstrated the importance of accurate pathology. It’s difficult to obtain lesion tissues by biopsy or surgical resection for pathological examination of patients with multiple metastases, because of intracranial hypertension or cerebral haemorrhage. It is essential to establish a rapid and non-invasive imaging method for pathological classification of BM [7].

Contrast-enhanced magnetic resonance imaging (MRI) is the first choice for the screening, diagnosis, and treatment of BM patients. The contrast enhancement caused by media extravasation is attributed to the increased vascular permeability with the exchange of contrast media between different compartments since the capillary permeability of metastases is higher than that of healthy tissues [8]. However, the imaging manifestations of different types of BM on the traditional 3 min enhanced images are similar, thereby making the pathological types undistinguishable [9, 10]. Serres et al. [11] confirmed the difference in growth pattern and angiogenesis of metastases among different primary tumour types through mouse experiments, with metastases from lung cancer and melanoma showing poor and rich blood supply, respectively [12, 13]. The difference in the angiogenic ability of different pathological type metastases and that in the heterogeneity between tumour cells and blood vessels will cause different dynamic changes in the contrast media over time, which will be displayed on delayed images [14].

Hatzoglou et al. [15] have shown that dynamic contrast-enhanced (DCE) MR perfusion imaging can distinguish the pathological types of BM by blood supply, but the authors have reported an area under the curve (AUC) of only 0.659, short imaging time and low resolution and that the contrast agent cannot be fully diffused into the tissue space. However, owing to sufficient time, the multi-phase delayed enhancement allows the contrast medium to filter from the blood vessels into the tissue space, which can reflect the dynamic changes in the blood supply of different types of BM. Especially for metastases with less blood supply, higher cell density, and high interstitial pressure, this dynamic change is even more complex and important.

We have previously reported that the accuracy of BM display and boundary delineation can be improved by multi-phase delay enhancement [16]. However, traditional MR images can only reflect the macroscopic information of BM, and the dynamic changes generated by contrast agents in multi-phase delayed enhancement can be analysed at the microscopic level by radiomics. The difference in contrast media diffusion is significantly associated with the heterogeneity of tumour cell and blood vessel interaction, which may be related to the pathological types of BM, thus providing a reliable basis for the accurate classification of BM pathological types.

Further, the segmentation and classification of BM depend on tumour size and contrast compared with healthy brain tissue, especially for multiple or small volume BM, which is easily missed and may lead to wrong treatment decisions [17, 18]. Although the segmentation and classification accuracy of BM has reached 0.79 and 0.9, respectively, the dynamic changes in BM MR image information caused by delayed time might impact the automatic segmentation and classification and could potentially improve the accuracy [19]. Few studies have fully considered the impact of dynamic changes in segmentation and classification for establishing models.

This study prospectively collected different phases of BM-enhanced MR images at 1, 3, 5, 10, 18, and 20 min to analyse the diffusion rule of multi-phase delayed enhanced MR T1 image contrast media by radiomics and to establish an automatic classification and segmentation model of BM based on support vector machine (SVM) and DPN-Unet. The best single phase and combination of multi-phases for BM automatic segmentation and classification to establish the model were also identified.

## Materials and Methods

### Patient Data

A total of 189 patients with BM with 1047 lesions were enrolled from the Affiliated Cancer Hospital of Shandong First Medical University between January 2020 and October 2021, of whom 98 were male individuals with an average age of 60 (36–80) years and 93 female individuals with an average age of 57.8 (36–81) years. The primary tumour types included small-cell lung cancer (53), lung adenocarcinoma (83), lung squamous-cell cancer (22), breast cancer (19), oesophageal cancer (7), renal clear-cell carcinoma (2), rectal cancer (1), and diffuse large B-cell lymphoma (2). For all enrolled patients, the histological type of the primary malignancy was obtained. The study was approved by the review board of the Affiliated Cancer Hospital of Shandong First Medical University.

### Image Acquisition

All images were acquired using computed tomography (CT) and MR simulations. During CT simulation, the patient was supine, with their head fixed with a thermoplastic film, and a Philips Brilliance Big Bore CT positioning machine (Philips Healthcare, Best, Netherlands) was used to scan with a 3-mm layer thickness and 3-mm layer spacing. Following CT simulation, the patients were placed in the same position and fixed mode using a 6-channel flex coil in GE 3.0 T MR scanner (Discovery 750 W; GE Healthcare, Chicago, IL, USA), and T1 plain scan and multi-phase enhanced 3D T1-weighted gradient-echo brain volume images (T1 BRAVO) at 1, 3, 5, 10, 18, and 20 min were obtained after intravenous injection of gadolinium-containing contrast medium.

The scan parameters were set as follows: TR = 8.5 ms, TE = 3.2 ms, matrix = 256 × 256, FOV = 256 mm × 256 mm, slice thickness = 1 mm, and slice number = 50–52; the T1 acquisition time of one phase was limited to between 1 min 55 s and 1 min 58 s. Gadoteric acid meglumine salt was injected with a binocular high-pressure syringe system (MEDRAD^®^ Spectris Solaris EP, Bayer, Leverkusen, Germany) at a rate of 2 mL/s and dose of 0.2 mL/kg and flushed with 20 mL saline.

### Tumour Segmentation

Lesions were defined as moderate-to-low signal on unenhanced T1 and showed focal abnormal enhancement on T1-enhanced images with higher signal intensity than normal brain tissue [20]. All MR images were imported into MIM Maestro (7.1.7; MIM Software, Beachwood, OH, USA), and rigid fusion registration was performed on the images of the six phases. The BM lesions and normal brain tissue, which were the region of interest (ROI) on different phase MR images were manually counted by three radiologists.

### Automatic Segmentation Model Verification

Automatic delineation of BM was performed using DPN-UNet, a convolutional neural network (CNN). UNet is one of the most commonly used CNN-based target delineation models. Dpn-UNet, a segmentation network implemented by embedding, implants the block in a dual path network (DPN) into the encoding and decoding layers in UNet. The DPN, in turn, encodes the input image into several high-level abstract features and parameters.

**Fig. 1:**
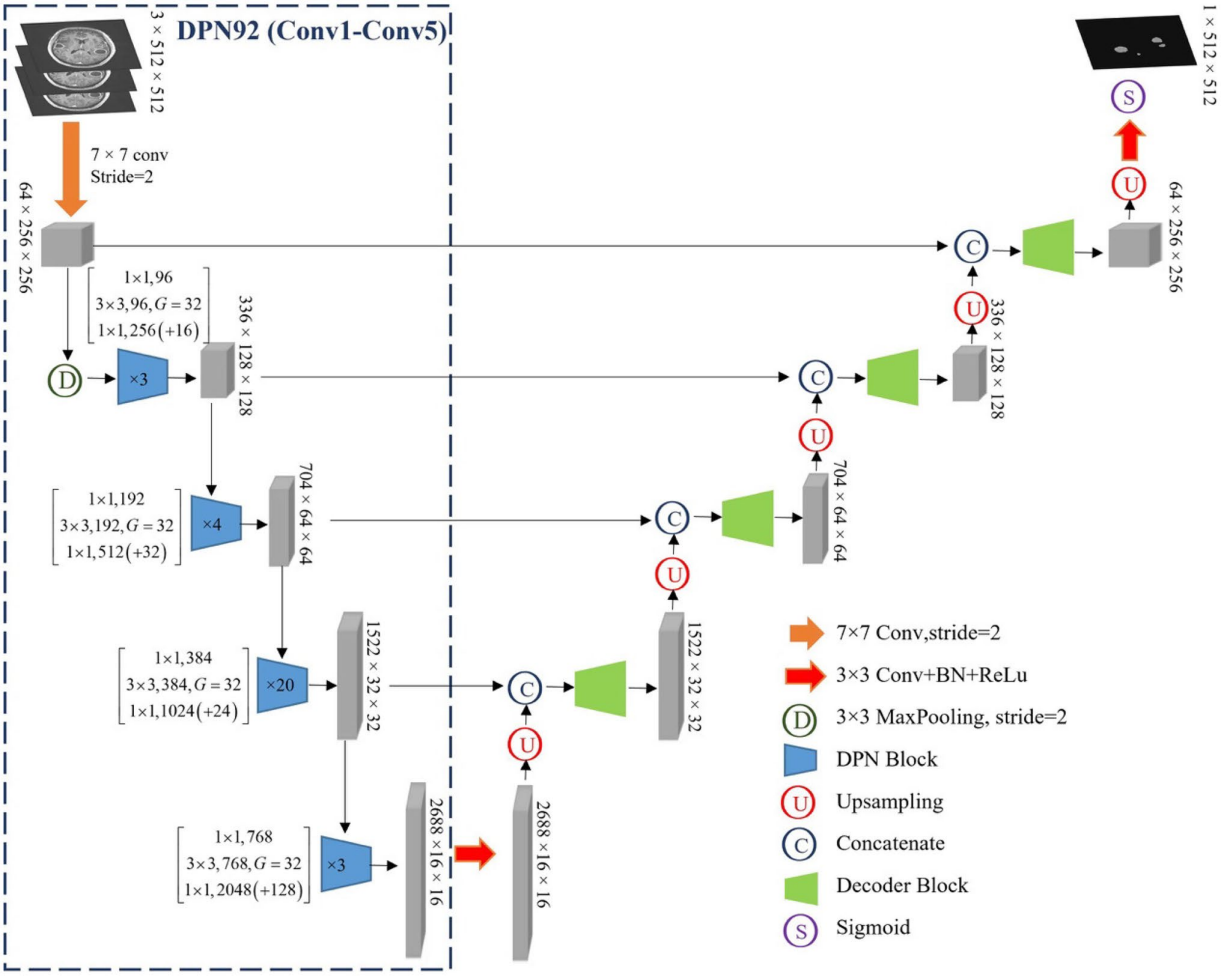
Dpn-UNet network structure diagram. DPN, dual path network

**Fig. 2:**
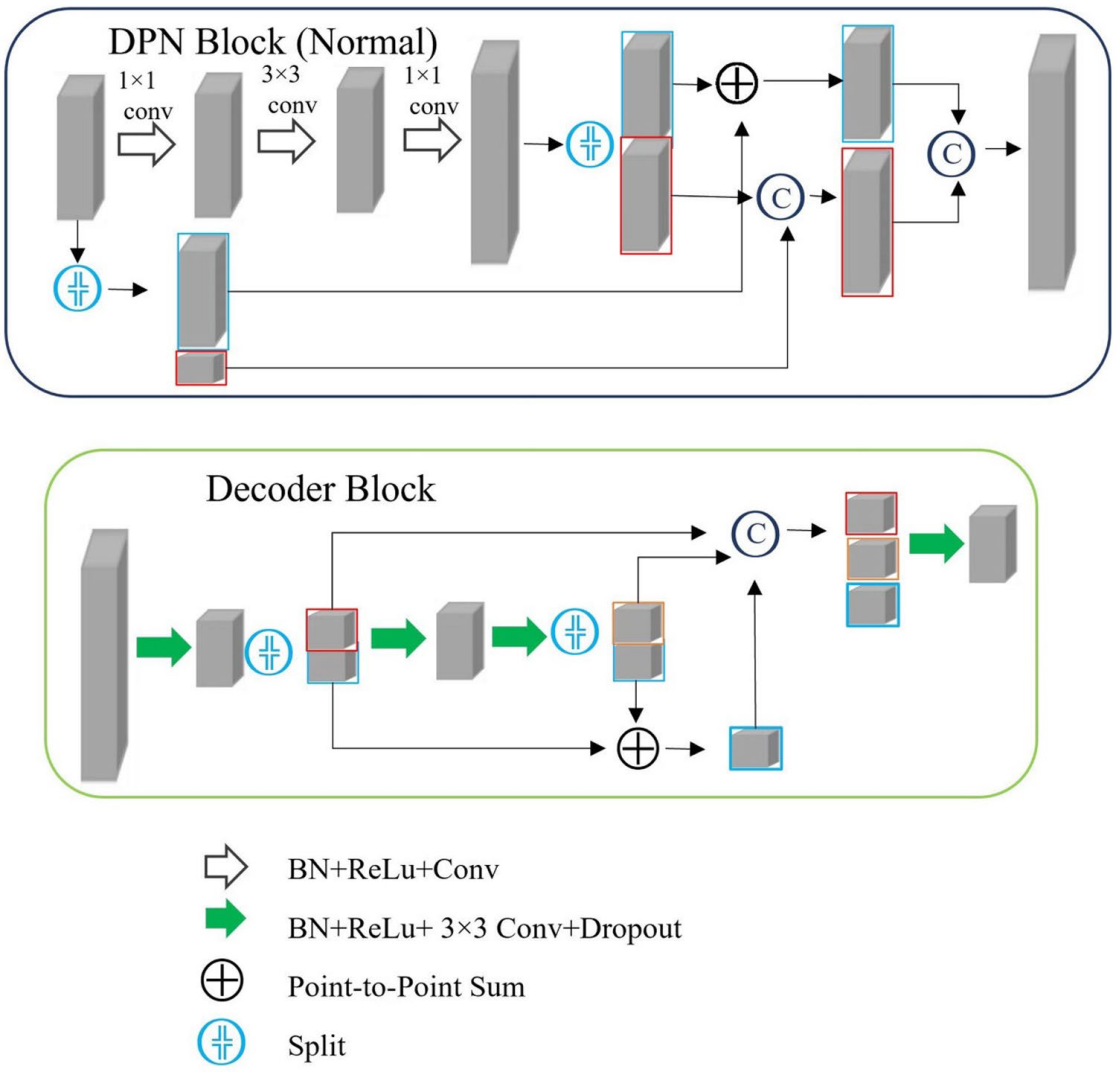
DPN block structure diagram

As shown in Figs. 1 and 2, the upper part is the image encoder using the DPN, the middle part is the decoder, and the lower part is the internal structure of the main module used by the decoder, which also draws lessons from the fusion of the ResNet and DenseNet module results of the DPN for up-sampling. Moreover, the implementation details of the loss function, learning rate, batch size, optimizer, and framework were set following our previous study [21].

### Extraction and Screening of BM Radiomics Features

**Fig. 3:**
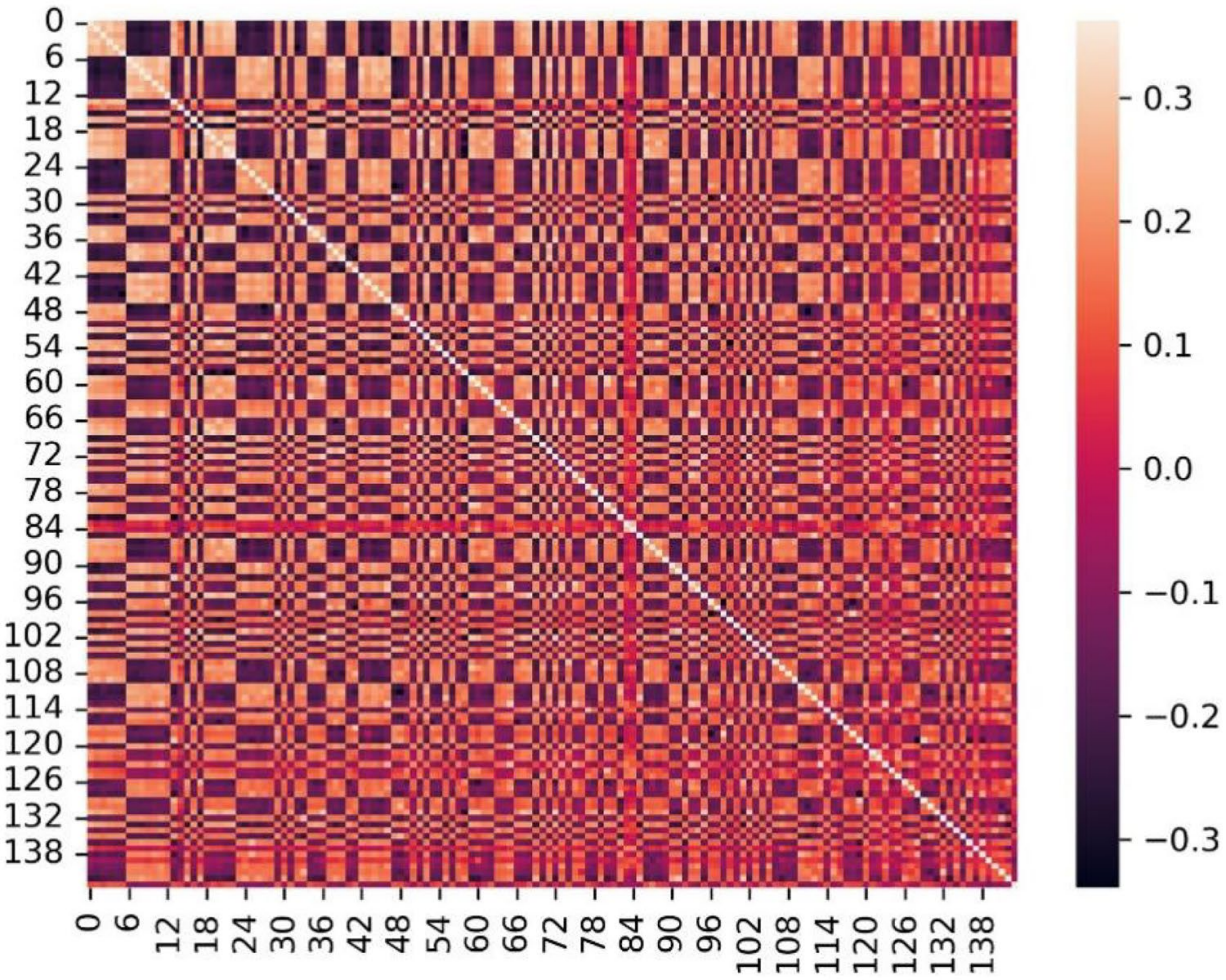
Thermal map showing the correlation coefficient among the 144 features screened out. The correlation coefficient from low to high corresponds to the colour band from deep to shallow, respectively. The maximum value is 0.3, indicating that these features are not correlated with each other

A total of 1781 initial radiomics features were extracted from each ROI using MATLAB. To improve the accuracy and calculation speed of the classification and regression analysis, we performed dimensionality reduction processing on radiomics features. In this study, the distribution rule of features in different phases was used, Pearson’s correlation coefficient (*R*) was used to calculate the change rule of features over time, and the statistical results of the correlation coefficients of different features with time were obtained by ranking them from the largest to the smallest. Simultaneously, considering the information redundancy between different features, we also calculated the correlation coefficient between the features and removed redundant features according to the level of the correlation coefficient. Simultaneously, to improve the execution efficiency of the machine learning algorithm, the intra-class variance of each feature itself was calculated, and the features with intra-class variance below 60 were removed. Finally, 144 radiomics features were obtained after screening for subsequent classification, which were analysed according to the correlation coefficient and their volatility over time. After calculating the correlation coefficients between all features and time, the following distribution rules were revealed: (1) The features show an obvious positive correlation with time, and (2) the features fluctuate with time without obvious rules (Fig. 3).

### Pathological Classification Model of BM

In this study, 176 patients (83 with lung adenocarcinoma, 53 with small-cell lung cancer, 22 with squamous-cell lung cancer, and 18 with breast ductal carcinoma in situ) were selected for the classification model that was established by applying SVM.

SVM is a supervised non-parametric statistical learning technique that does not make any assumptions about the underlying data distribution [22]. In its original formulation, the method provides a set of labelled data instances and aims to find a hyperplane that divides the dataset into a discrete predefined number of class examples in a manner consistent with the training model as follows:

1 $$f\left(x\right)=\sum\limits_{i\,=\,1}^{m}{\alpha }_{i}{y}_{i}K\left(x, {x}_{i}\right)+\frac{1}{m}\sum\limits_{j\,=\,1}^{m}({y}_{i}-\sum\limits_{i\,=\,1}^{m}{\alpha }_{i}{y}_{i}{x}_{i}{x}_{j})$$

where (${x}_{i}, {y}_{i}$) is the sample point set, *K* is the kernel function, and ${\mathrm{\alpha }}_{\mathrm{i}}$ is the constraint parameter.

The random forest classifier (RF) is an algorithm that integrates multiple trees using ensemble learning theory. The basic unit is a decision tree. After random sampling of the original training data, each decision tree in the forest is used to judge the unlabelled samples, and the majority vote of all decision trees is applied to predict the unlabelled sample category [23]. This method has a relatively low tendency for overfitting, and the model is as follows:

2 $$RF\left(x\right)=\frac{1}{B}\sum\limits_{i\,=\,1}^{B}{T}_{i,{z}_{i}}(x)$$

where $RF\left(x\right)$ represents the prediction value of the random forest for the sample $x$, B represents a total of B trees, ${z}_{i}$ represents the training set used by the i tree, and ${T}_{i}$ represents the learner of the i^th^ tree.

### Classification Model Verification

SVM and RF methods from the SKLEARN machine learning package in the Python library were used, combined with radiomics feature vectors, and regression prediction analysis was performed on pathological types [24]. During training, the training/test data ratio was 7/3. To verify the significance of the introduction of multi-phase data and improvement of the accuracy of the classification results brought by the combination of multi-phase data, this study used the data of 1, 3, 5, 10, 18, and 20 min alone or two-two, three-three, four-four, or five-five combination methods, put into a machine learning classifier for model training and verification.

### Statistical Analyses

SPSS (version 26.0; IBM, Armonk, NY, USA) statistical software was used to conduct a paired-samples *t-*test for the maximum and minimum mean signal intensities of metastases and Wilcoxon rank-sum test for the average dice similarity coefficient (DSC) between different phases. Statistical significance was established at *P* < 0.05.

## Results

### Analysis of Tumour Grey level Changes at Different Enhancement Phases

**Fig. 4:**
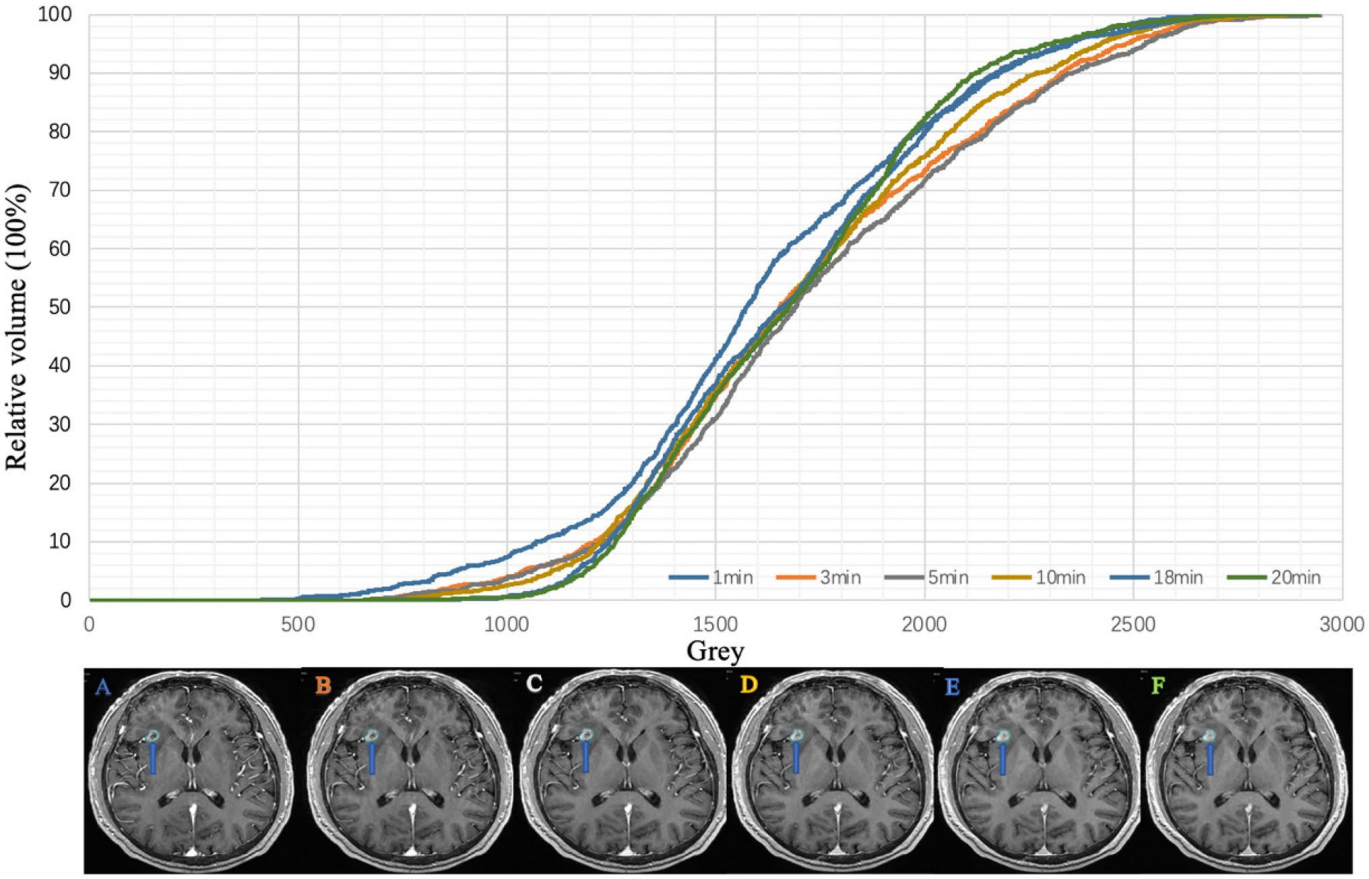
Grey histogram of patients with lung adenocarcinoma in six phases: **A**, **B**, **C**, **D**, **E**, and **F** correspond to 1-, 3-, 5-, 10-, 18-, and 20-min images, respectively

**Fig. 5:**
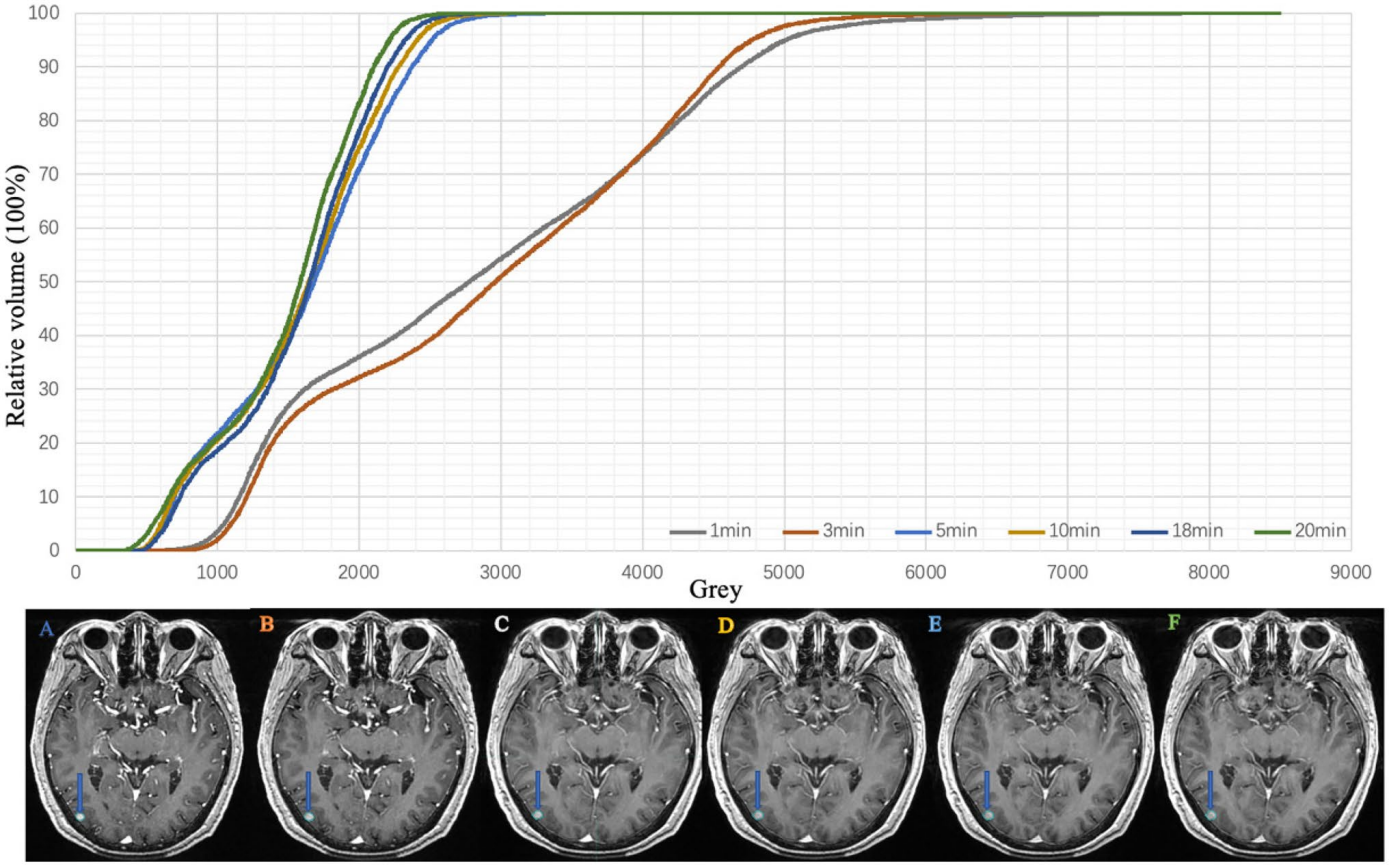
Grey histogram of patients with lung squamous cell carcinoma in six phases: **A**, **B**, **C**, **D**, **E**, and **F** correspond to 1-, 3-, 5-, 10-, 18-, and 20-min images, respectively

The mean grey level of all metastases in 189 patients were 1403.0, 1467.0, 1457.9, 1419.4, 1387.6, and 1375.5 at 1, 3, 5, 10, 18, and 20 min, respectively, and the peak grey level was observed at 3 min. Compared with that at 3 min, the grey level at 1, 5, 10, 18, and 20 min decreased by 4.6%, 0.6%, 3.4%, 5.7%, and 6.7%, respectively, and the mean grey level showed a downward trend as time progressed. There was a significant difference between the maximum and minimum grey level (*P* < 0.05), as shown in Figs. 4 and 5.

### Analysis of Radiomics Features in Different Phases

In total, 22 (15.3%) of the 144 features showed a significant positive correlation with time (*R* > 0.7). The highest correlation coefficient was 0.82 for wavelet-HLH-GlDm-graylevelnonclothes. The other 122 features fluctuated with time, and their correlation coefficients were in the range of 0.3–0.7 (Table 1).

**Table 1:** Correlation coefficients of radiomics features with time (*R* > 0.7)

**Feature name**	**Correlation coefficient**
**wavelet-HLH-gldm-GrayLevelNonUniformity**	0.8234
**wavelet-LHH-gldm-GrayLevelNonUniformity**	0.8226
**wavelet-LHL-gldm-GrayLevelNonUniformity**	0.8121
**gradient-gldm-SmallDependenceHighGrayLevelEmphasis**	0.7834
**log-sigma-1-mm-3D-gldm-GrayLevelNonUniformity**	0.7833
**logarithm-gldm-GrayLevelNonUniformity**	0.783
**wavelet-LLH-gldm-GrayLevelNonUniformity**	0.7681
**wavelet-LHH-gldm-GrayLevelVariance**	0.7528
**gradient-firstorder-TotalEnergy**	0.7511
**wavelet-LHH-firstorder-10Percentile**	0.7409

**Fig. 6:**
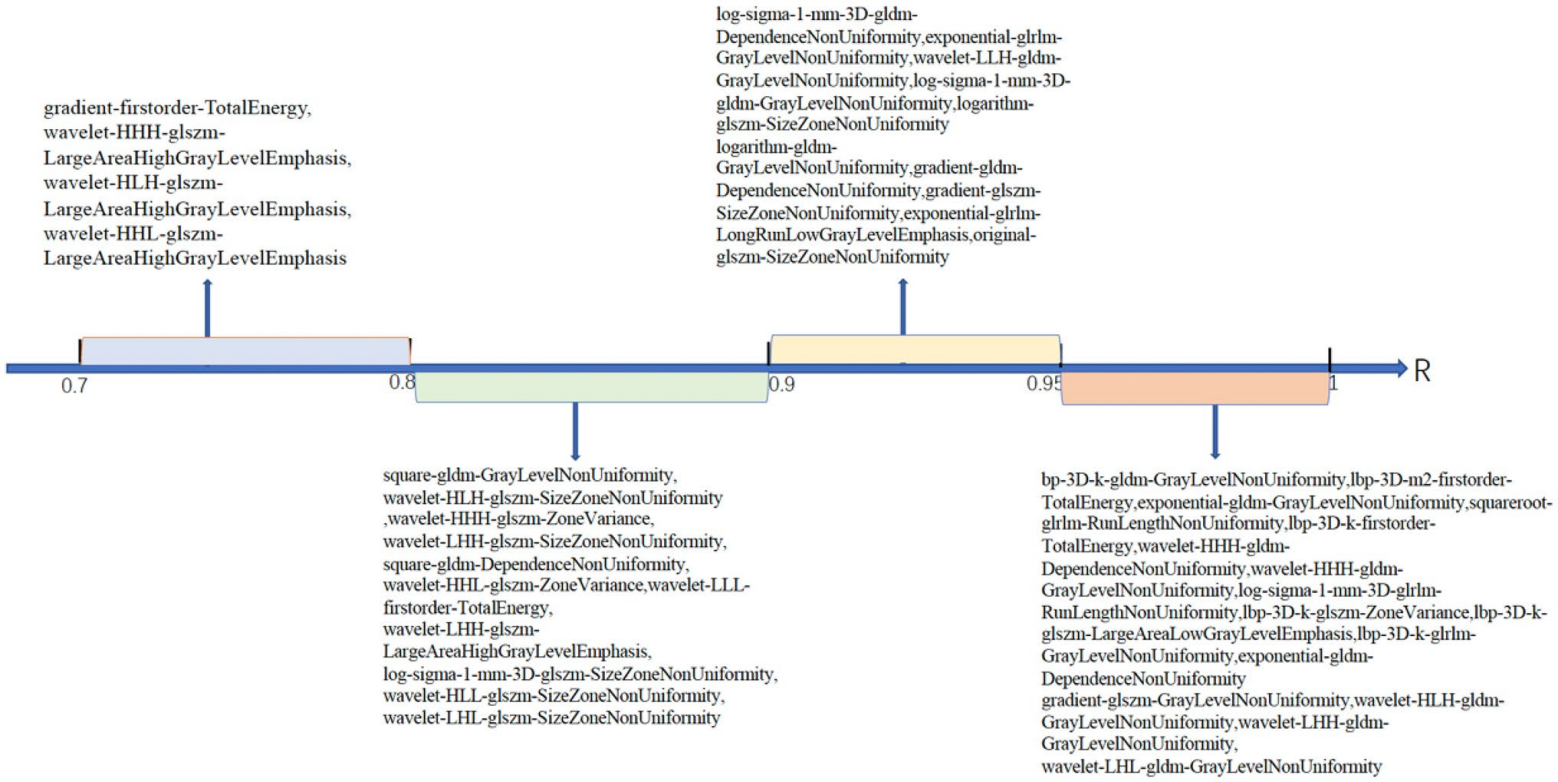
Correlation coefficients of radiomics features with volume change (0.7 < *R* < 1). Each colour represents a different range of correlation coefficients

Among the 144 features, 41 were positively correlated with volume (all *R* > 0.7). The highest correlation coefficient was 0.99 for LBP-3D-K-GlDM-graylevelnonclothes. There was a strong correlation between the volume and features (Fig. 6).

### Effect of Different Enhanced Phases MR Images on Automatic Segmentation of BM

The average tumour volume of the six phases, ranging from 0.03–53.2 cm^3^, was used as the ROI volume index. Patients were randomly divided into training (*n* = 140) and test sets (*n* = 49). In the training set, the average DSCs at 1, 3, 5, 10, 18, and 20 min were 0.9, 0.9, 0.91, 0.92, 0.91, and 0.91, respectively. The highest DSC was at 10 min; the DSCs first increased and then decreased as the enhancement time prolonged.

**Fig. 7:**
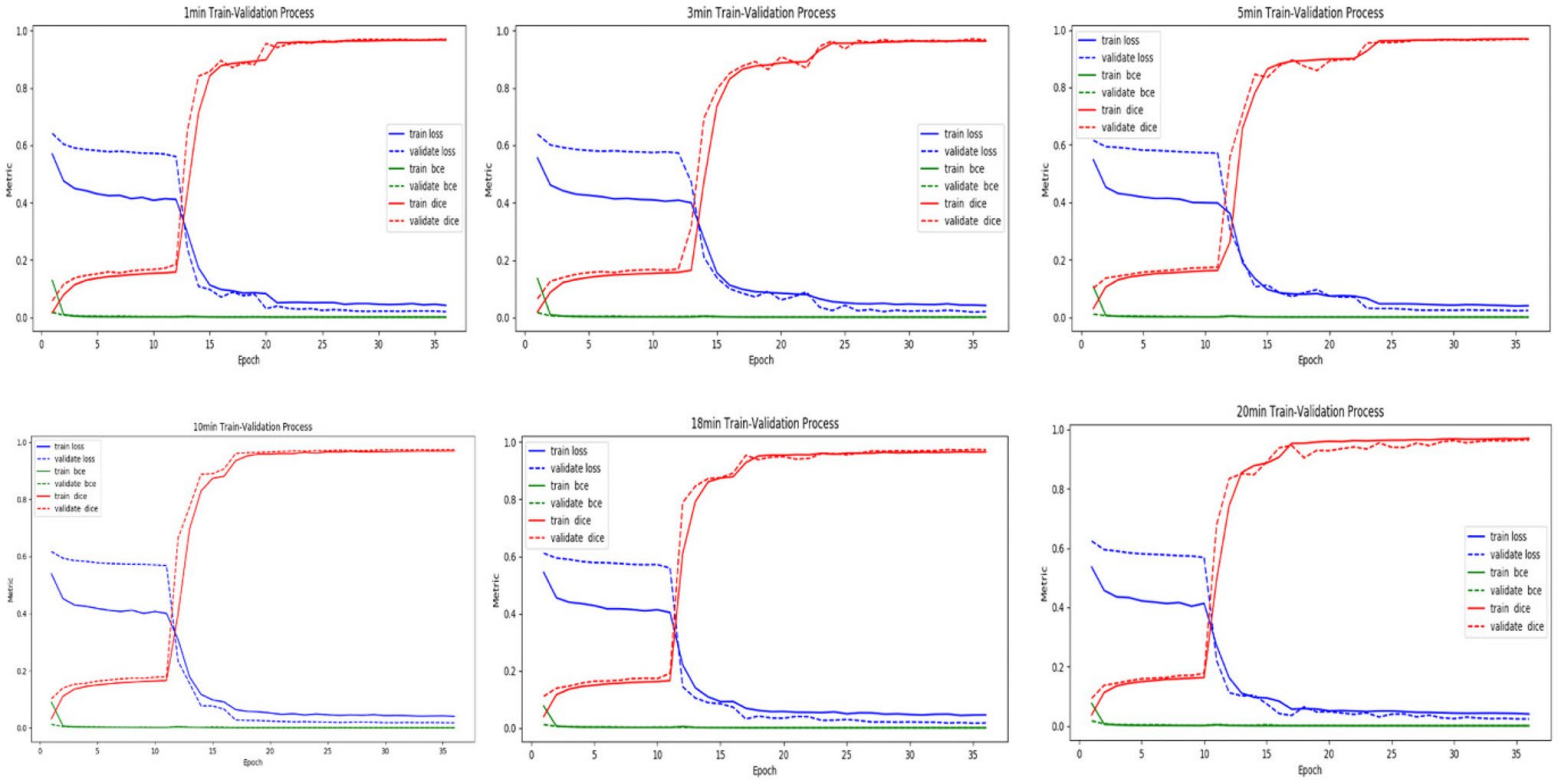
Changes in train loss, validate loss, train bce, validate bce, train dice, and validate dice in six phases

Conversely, in the test set, the average DSCs at 1, 3, 5, 10, 18, and 20 min were 0.78, 0.79, 0.81, 0.82, 0.82, and 0.81, respectively. The highest DSC was observed at 10 and 18 min, and the DSC first increased and then decreased as the enhancement time prolonged. Compared with that at 3 min, the automatic segmentation accuracies at 5, 10, 18, and 20 min were improved by 2.5%, 3.8%, 3.8%, and 2.5%, respectively, while the accuracies at 10 and 18 min were not significantly different (*P* > 0.05) (Table 2 and Fig. 7).

**Table 2:** Average dice coefficients of brain metastasis segmentation at different phases

Category	Delay phase					
	1 min	3 min	5 min	10 min	18 min	20 min
Test set	0.78 ± 0.13	0.79 ± 0.12	0.81 ± 0.11	0.82* ± 0.10	0.82 ± 0.10	0.81 ± 0.11
Training set	0.90 ± 0.07	0.90 ± 0.06	0.91 ± 0.05	0.92* ± 0.05	0.91 ± 0.07	0.91 ± 0.06

*Present for the highest DSCs

### Influence of Different Enhanced MR Phases on BM Pathological Classification

For the primary tumour classification of BM, when creating an SVM classifier for classification prediction, the accuracy reached 0.87, while the classification accuracy of RF only was 0.76.

### Single-Phase Classification Prediction

When different phases were independently selected for BM classification prediction, the AUCs at 1, 3, 5, 10, 18, and 20 min were 0.585, 0.635, 0.641, 0.674, 0.614, and 0.653, respectively. The AUC at 10 min was the highest, consistent with segmentation. The AUC first increased up to 10 min and then decreased. Compared with that at 10 min, the decrease in the AUCs at 1, 3, 5, 18, and 20 min reached − 15.2%, − 6.1%, − 5.1%, − 9.8%, and − 3.2%, respectively (Table 3).

**Table 3:** AUCs and accuracies of independent phases for brain metastasis classification

**Single phase**	**AUC**	**Accuracy**
**1 min** **3 min** **5 min** **10 min** **18 min** **20 min**	0.5855 0.6359 0.6410 0.6743 0.6147 0.6533	0.5868 0.6169 0.6477 0.6242 0.6296 0.6464

*AUC* area under the curve

### Classification Prediction Applying Combination with Multi-phase MR Images

**Fig. 8:**
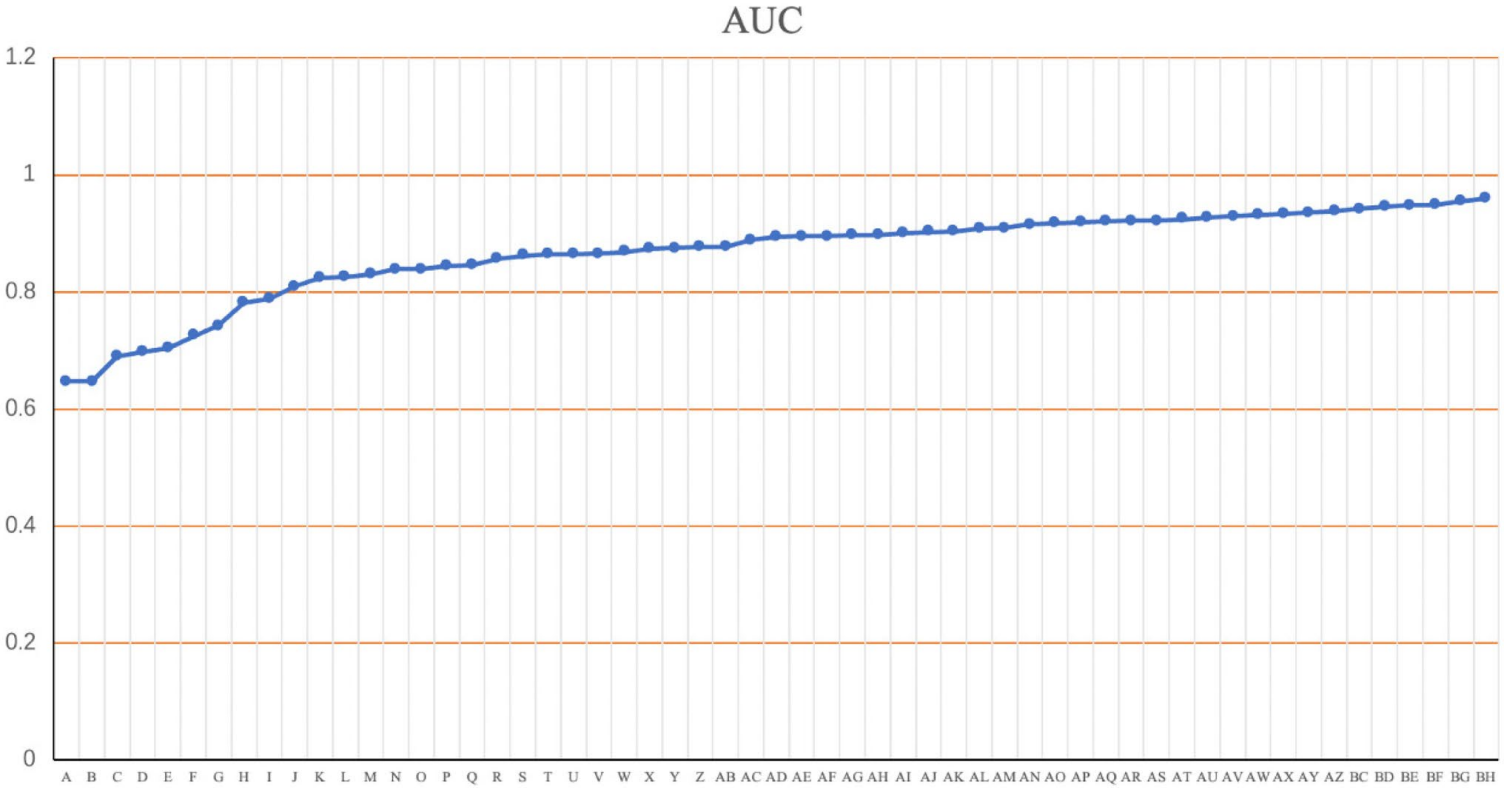
From the combination of two phases to six phases, the AUC showed an increasing trend as the number of phases increased. Two phases: A: 1 and 20 min; B: 3 and 5 min; C: 1 and 18 min; D: 3 and 18 min; E: 3 and 20 min; F: 5 and 20 min; G: 1 and 10 min; H: 5 and 18 min; I: 3 and 10 min; J: 1 and 5 min; K: 5 and 10 min; L: 1 and 3 min; M: 10 and 18 min; N: 10 and 20 min; O: 18 and 20 min. Three phases: P: 1, 5 and 20 min; Q: 1, 3 and 20 min; R: 1, 10 and 20 min; S: 1, 18 and 20 min; T: 1, 3 and 18 min; U: 5, 18, and 20 min; V: 3, 5, and 20 min; W: 1, 5, and 18 min; X: 3, 5, and 18 min; Y: 3, 10, and 20 min; Z: 1, 10, and 18 min; AB: 3, 18 and 20 min; AC: 3, 10, and 18 min; AC: 1, 3, and 10 min; AD: 5, 10, and 20 min; AE: 5, 10, and 18 min; AF: 3, 5, and 10 min; AG: 10, 18, and 20 min; AH: 1, 3, and 5 min. Four phases: AI: 1, 10, 18 and 20 min; AJ: 1, 5, 10, and 20 min; AK: 1, 5, and 10 min; AL: 1, 5, 18, and 20 min; AM: 1, 3, 5, and 18 min; AN: 1, 3, 18, and 20 min; AO: 1, 5, 10, and 18 min; AP: 1, 3, 10, and 20 min; AQ: 1, 3, 10, and 18 min; AR: 3, 5, 18, and 20 min; AS: 1, 3, 5, and 20 min; AT: 3, 5, 10, and 20 min; AU: 3, 10, 18, and 20 min. Five phases: AV: 1, 5, 10, 18, and 20 min; AW: 1, 3, 5 10, and 20 min; AX: 5, 10, 18, and 20 min; AY: 1, 3, 5, 18, and 20 min; AZ: 3, 5, 10, and 18 min; BC: 1, 3, 10, 18, and 20 min; BD: 1, 3, 5, 10, and 18 min; BE: 1, 3, 5, and 10 min; BF: 3, 5, 10, 18, and 20 min. Six phases: BG: 1, 3, 5, 10, 18, and 20 min

Compared with that of the single-phase classification model, the AUC of the multi-phase image combination model was significantly improved and showed a regularity in which the AUC was higher as more phases were introduced into the model. Among them, the AUC of the six-phase combination model was the highest, which reached 0.9596. The AUCs of the five-phase combination model were 0.9482, 0.9337, 0.9379, 0.9454, 0.9316, and 0.9545, and the combined model with the highest AUC was 3, 5, 10, 18, and 20 min. The AUCs of the four-phase combination model were in the range of 0.8951–0.9486, and the combined model with the highest AUC was 1, 3, 5, and 10 min. The AUCs of the three-phase combination model were in the range of 0.8234–0.9275, and the combined model with the highest AUC was 1, 3, and 5 min. The AUCs of the two-phase combination model were in the range of 0.6469–0.9099, and the combined model with the highest AUC was 18 and 20 min. Using 5 and 10 min as data for classification showed a superior classification accuracy to the overall value. Compared with the optimal single-phase accuracy and AUC, the multi-phase AUC was improved by 42.3% (Fig. 8).

## Discussion

With the continuous increase in the incidence of BM, accurate tumour staging and histopathological identification of intracranial metastatic diseases are essential [25]. The pathological type of primitive cancer cells determines the different properties of BM growth and response to treatment, with angiogenesis being the key factor for the growth and metastasis of BM [26]. Therefore, how the dynamic changes in BM blood supply are presented can better clarify the pathological classification.

Contrast-enhanced MRI revealed that vascular permeability is related to tumour aggressiveness, and a marked increase in signal intensity in contrast-enhanced areas is a diagnostic criterion for BM [27]. There are significant differences in the heterogeneity of tumour cells and blood vessels following metastasis in different pathological types of primary tumours, and the penetration of contrast media into a tumour is time dependent. Therefore, we propose that multi-phase delayed enhancement can reflect the dynamic change in the contrast medium dispersion in BM. However, previous studies on MR radiomics for BM pathology prediction have only used conventional static timing of 3 or 5 min [28].

Tumour pathological types can be identified using dynamic susceptibility contrast MRI; however, if both are hypovascular types, their perfusion imaging features will overlap when the imaging time is short [29]. Based on the macroscopic manifestations of delayed imaging, we applied radiomics to microscopically analyse such dynamic changes and to successfully identify BM pathological types.

Changes in BM signal intensity and volume with delayed enhancement time will inevitably affect the extraction and analysis of radiomics features. In this study, the radiomics features were strongly correlated (with correlation coefficients as high as 0.82 and 0.99) with enhancement delay time and BM volume. Radiomics features can accurately predict the pathological type of tumours. Kniep et al. [30] confirmed that quantitative MRI radiomics features predict different pathological types of BM with an AUC of 0.9; however, in this study, the best AUC was obtained with the combination of six phases, reaching 0.9596, which is a better outcome.

In the automatic classification of BM, the 10-min AUC was the highest on single-phase MRI, but it was only 0.67, while the AUC of a multi-phase combined application can reach 0.9596. This indicates that a multi-phase combination can better distinguish pathological types of BM than a single phase. Among them, the best combination was the six-phase combination (1, 3, 5, 10, 18, and 20 min), which was 42.3% higher than the best single phase. The main reason for this is that the dynamic change in the contrast agent permeating from blood vessels to tissue can better reflect the pathological type of tumour. Among other combinations, the result that the four-phase combination was better than both the three- and two-phase combinations also confirms this hypothesis. In the multi-phase combination of each group, the public phases were 5 and 10 min, indicating that tumour imaging at 5–10 min of delayed enhancement is more advantageous for BM classification. Béresová et al. [31] found that BM volume affects the reliability of the classification model, confirming that dynamic changes in the BM volume in different enhancement phases can also affect the classification results. However, Kang et al. [32] confirmed that the tumour volume at 10 min is significantly larger than that at 1 min, and this study suggests that a single phase of 10 min or a combination of phases of 10 min is more reliable for BM classification prediction.

Results from studies using artificial intelligence technology for the automatic segmentation of BM based on traditional enhanced phases of 3 or 5 min are inconsistent. The average DSC obtained by Grøvik et al. [33] was 0.79, consistent with the traditional 3-min DSC in our study.

However, by applying the traditional Dpn-UNet model to different delay enhancement phases, our results showed that the segmentation accuracy is the highest at 10 min, and the average DSC first increases and then decreases with the enhancement time delay. Compared with the conventional 3-min DSC, the average 10- or 18-min DSC increased by 3.8%. This was because the contrast agent did not fully penetrate the tumour at 3 min, and the contrast agent entered a dynamic equilibrium state with a delay of 10 min; thus, the signal difference between the normal tissue and tumour is more stable. Kushnirsky et al. [34] found that 10–15 min of tumour imaging reached the best state, consistent with our study. Qiu et al. [35] studied the optimal phase for delineating the edge of gliomas in mice based on delayed enhancement MRI and confirmed that the enhancement phase has a significant effect on tumour edge display, which can lead to differences in segmentation.

Reportedly, the results of automatic segmentation and classification of BM significantly changed with the delayed enhancement phase, confirming that the dynamic changes in BM during multi-phase delayed enhancement impact the performance of BM segmentation and classification. The SVM and DPN-Unet models applied in this study have a mature algorithm, stable performance, and clinical application, thereby reducing the differences in the model itself [36]. This study has some limitations. The selected data were all images of patients before radiotherapy, while the sample size was limited; however, the impact of dynamic changes in multi-phase enhancement on the performance of artificial intelligence segmentation and classification has been confirmed.

## Conclusion

This study observed the effect of multi-phase delayed enhancement on the accuracy of BM segmentation and classification, confirming the limitations of the traditional enhanced phase for artificial intelligence in BM segmentation and classification. However, the dynamic changes in the contrast media diffusion in BM can be reflected by multi-phase delayed enhancement based on radiomics, which can more objectively reflect the pathological types of BM and significantly improve the accuracy of BM segmentation and classification. The contrast-enhanced images at 10 min were the best for segmentation and classification by applying single-phase images. The six-phase image combination can achieve the highest classification accuracy.

## Data Availability

The datasets generated and analysed during this study are available from the corresponding author on reasonable request.

## Abbreviations

BM: Brain metastases
SVM: Support vector machine
DSCs: Dice similarity coefficients
AUCs: Areas under the curve
DCE: Dynamic contrast-enhanced
ROI: Region of interest
CNN: Convolutional neural network
DPN: Dual path network
RF: Random forest
DSC: Dynamic susceptibility contrast
MRI: Magnetic resonance imaging
AI: Artificial intelligence
